# Target trial emulation of aspirin after diagnosis of colorectal polyps

**DOI:** 10.1007/s10654-023-01024-1

**Published:** 2023-06-15

**Authors:** Louise Emilsson, Mingyang Song, Jonas F. Ludvigsson

**Affiliations:** 1 Department of General Practice, Institute of Health and Society, University of Oslo, Oslo, Norway; 2 Vårdcentralen Värmlands Nysäter and Centre for Clinical Research, County Council of Värmland, Värmland, Sweden; 3 Department of Medical Epidemiology and Biostatistics, Karolinska Institutet, Solna, Sweden; 4 Faculty of Medicine and Health, Örebro University, Örebro, Sweden; 5 Departments of Epidemiology and Nutrition, Harvard T.H. Chan School of Public Health, Boston, MA USA; 6 Clinical and Translational Epidemiology Unit, Massachusetts General Hospital and Harvard Medical School, Boston, MA USA; 7 Division of Gastroenterology, Massachusetts General Hospital and Harvard Medical School, Boston, MA USA; 8 Department of Pediatrics, Örebro University Hospital, Örebro, Sweden; 9 Celiac Disease Center, Department of Medicine, Columbia University Medical Center, New York, NY USA

**Keywords:** Cancer prevention, Precision medicine, Target trial emulation, Causal inference

## Abstract

**Backgound and Aims:**

Previous research on the potential chemoprotective effect of aspirin for colorectal cancer (CRC) shows conflicting results. We aimed to emulate a trial of aspirin intiation in individuals with incident polyps.

**Methods:**

We identified individuals registered with their first colorectal polyp in the nationwide gastrointestinal ESPRESSO histopathology cohort in Sweden. Individuals aged 45–79 years diagnosed with colorectal polyps 2006–2016 in Sweden without CRC or contraindications for preventive aspirin (cerebrovascular disease, heart failure, aortic aneurysms, pulmonary emboli, myocardial infarction, gastric ulcer, dementia, liver cirrhosis, or any other metastatic cancer) registered until the month of first polyp detection were eligible. Using duplication and inverse probability weighting, we emulated a target trial of aspirin initiation within 2 years of initial polyp detection. The main outcome measures were incident CRC, CRC mortality and all-cause mortality registered until 2019.

**Results:**

Of 31,633 individuals meeting our inclusion criteria, 1716 (5%) initiated aspirin within 2 years of colon polyp diagnosis. Median follow-up was 8.07 years. The 10-year cumulative incidence in initiators versus non-initiators was 6% versus 8% for CRC incidence, 1% versus 1% for CRC mortality and 21% versus 18% for all-cause mortality. The corresponding hazard ratios were 0.88 (95% confidence interval, 95%CI = 0.86–0.90), 0.90 (95%CI = 0.75–1.06) and 1.18 (95%CI = 1.12–1.24).

**Conclusion:**

Aspirin initiation in individuals with polyp removal was linked to 2% lower cumulative incidence of CRC after 10 years but did not alter CRC mortality. We also observed a 4% increased risk difference of all-cause mortality at 10 years after the initiation of aspirin.

**Supplementary Information:**

The online version contains supplementary material available at 10.1007/s10654-023-01024-1.

## Introduction

Colorectal cancer (CRC) is the second leading cause of cancer deaths worldwide [1]. Removing precursor lesions [2, 3] can reduce the risk of CRC incidence and mortality. However, after the removal of high-risk polyps, individuals still have an increased risk of future CRC compared to individuals without colorectal polyps [4]. Based on the extrapolation of cardiovascular prevention trials [5] and observational studies, daily aspirin intake has been proposed to prevent the development of CRC. The effectiveness has been comparable to endoscopic or fecal blood screening [6]. In 2016 the United States Preventive Services Task Force (USPSTF) advocated low-dose aspirin treatment for the primary prevention of cardiovascular disease (CVD) and CRC in adults aged 50–59 years [7]. However, a recently published randomized trial in healthy people age > 65—the ASPREE trial—was prematurely terminated as it showed increased all-cause mortality^8^ and, more specifically, increased CRC mortality [8] in aspirin users. Therefore, the USPSTF revisited the issue of aspirin prevention and published a draft evidence review in October 2021 [9]. The review concluded that current evidence (data from primary CVD prevention populations [9] and longer-term follow-up data from the Women's Health Study, WHS) did not support the claim that low-dose aspirin reduces CRC incidence or mortality. Contrary to the USPSTF review, the American College of Gastroenterology recommends aspirin for CRC prevention in individuals aged 50–69 years with a cardiovascular disease risk of ≥ 10% over the next 10 years but no increased risk of bleeding [10]. Outcome discrepancy (from a large reduction to increased risk of CRC and CRC mortality) is likely explained by differences in study populations (effect modification), study design (RCT vs. observational extrapolation of RCT after the end of study vs. observational studies) and lack of assessment of competing risk in selected populations. To enhance knowledge on aspirin in CRC prevention, we emulated a study of aspirin initiation in a selected group most likely to benefit from CRC prevention; individuals with newly diagnosed colorectal polyps, where prevention could also be thought of as “secondary” given the increased risk of CRC in this patient group.

## Materials and methods

### Study population

Using the unique personal identity number assigned to all Swedish residents [11], we identified individuals with their first diagnosed colorectal polyps [4] from the nationwide ESPRESSO cohort (Epidemiology Strengthened by histoPathology Reports in Sweden) that contains biopsy report data from all 28 pathology departments in Sweden between 1965 and 2017 [12]. Histopathologic findings defined by codes of morphology (Swedish modification of the Systematized Nomenclature of Medicine [SNOMED] coding system) and topography, T67 (colon) and T68 (rectum), in combination with any relevant SNOMED codes for adenomas or validated [13] free text search for serrated polyps, were used to identify individuals with polyps. Thus, the baseline population was the same as the cases in our earlier study [4].

### Target trial

*Inclusion criteria* were a registered incident colorectal polyp as defined above, age 45–79 years (polyps occurring before 45 years are likely hereditary and individuals aged over 80 years are unlikely to benefit from CRC prevention because of advanced age, not least since CRC develops over many years) and being diagnosed from 1 July 2006 to 31 Dec 2016 to ensure ≥ 1 year of no previous prescription of anticoagulants as registered in the Prescribed Drug Register (Nationwide in Sweden from 1 July 2005) prior to study entry and to allow for ≥ 3 full years of follow-up before the administrative end of the follow-up (31 Dec 2019). *Exclusion criteria* were (I) previously registered prescription of either aspirin, warfarin or direct oral anticoagulant (DOAC) (i.e. ATC codes B01AC06 for Aspirin and B01AF, B01AE07 and B01AA03 for other anticoagulants) before the date of first colorectal polyp diagnosis. Individuals already on aspirin cannot benefit from initiation as they are already prevalent users and would not be eligible for a real-world trial and those on other anticoagulants would not be eligible because of the risk of bleeding complications. (II) individuals with existing CRC, dementia, metastasized malignancy, hemorrhagic stroke, gastric ulcer, aortic aneurysms, liver cirrhosis (contraindications for preventive aspirin initiation), pulmonary emboli (indication for DOAC or warfarin), myocardial infarction and cerebrovascular disease including TIA (transient ischemic attack)(due to the previous indication for aspirin initiation without actual initiation) registered up until and including the month of first polyp detection. We also excluded (III) those with any previous record of colorectal polyps. Because of the study design, we also had to exclude (IV) all individuals with follow-up ending during the same month as the detection of polyps. Follow-up started at the month of polyp detection and ended at event, death, emigration or administrative end of follow-up (31 Dec 2019), whichever occurred first (Supplementary table 3).

### Covariates

For all participants, we extracted a Charlson score [14] (0, 1, 2, 3, 4, ≥ 5), number of ever pre-baseline registered visits in the Swedish Patient Registry (continuous), any diabetes, heart failure, malignancies other than CRC, diagnosis of chronic obstructive pulmonary disease (COPD, proxy for heavy smoking), year of study entry, age, sex, number of diagnosed colorectal polyps (1, 2, ≥ 3) and educational attainment (≤ 9, 10–12, > 12 years and missing) at baseline. We also extracted the following time-varying covariates (coded as 0 until the first month of occurrence and thereafter always 1): ischemic stroke, bleeding stroke, myocardial infarction, angina pectoris, liver disease, dementia, ulcer, TIA, heart failure, COPD, diabetes, pulmonary emboli, aneurysm, other malignancy and metastatic malignancy for inclusion in our inverse probability weight (IPW) model for aspirin initiation. Initiation of aspirin was defined as the first prescription of ≥ 14 defined daily doses of aspirin of ATC code B01AC06. This definition is due to the Swedish dispensing system for individuals in which multiple medications delivered in packages (Swedish APODOS) iterated every 14 days. In Sweden, aspirin prescription constitutes > 95% of all use, with < 1% sold over the counter (at any dose) and the remaining ~ 4% is derived from acute care settings[15, 16]. Relevant codes are listed in the appendix.

### Emulating the target trial

Duplication and IPW methodology similar to previous studies [17] were used to emulate a trial in which all individuals who met our criteria for the target trial were duplicated in the dataset, and each of the replicates were assigned to one of the two treatment strategies: initiate aspirin (75 or 160 mg) anytime within 2 years of diagnosis of colorectal polyp or not to initiate aspirin within the first 2 years of colorectal polyp diagnosis.

The duplicates assigned to (1) aspirin initiation were artificially censored at month 24 if they had not initiated aspirin by then. The duplicates assigned to (2) no initiation of aspirin within the first 2 years were artificially censored at the actual month of aspirin initiation during month 1–24. However, initiation after 2 years did not lead to artificial censoring.

### Statistical analysis

The number of individuals initiating aspirin within 2 years from polyp detection is presented according to sex (female, male), age (45–54, 55–64, 65–74, 75–79), year of first colorectal polyp diagnosis (2006–2009, 2010–2012, 2013–2016) and educational attainment (≤ 9, 10–12, > 12 years and missing). Incidence rates of CRC mortality per 1000 person-years were calculated according to the status of aspirin initiation at 2 years and baseline subgroups. Hence, these incidence rates include some immortal time ranging from 1 to 24 months for those who initiated aspirin but are displayed to illustrate the crude number of events and incidence rates in the two groups. For the outcome of CRC incidence, we therefore present events for initiators according to their initiation status at 24 months rather than at the time of incident CRC diagnosis. However, the full trial emulation estimates are entirely free of immortal time bias.

We used pooled logistic regression for each outcome (CRC, CRC mortality, all-cause mortality), including the indicator of initiation (initiation of aspirin vs. no initiation of aspirin), month of follow-up (linear and quadratic terms), age (linear and quadratic terms), number of previous visits ever registered before study entry (linear and quadratic terms) and all baseline variables listed above as categorical variables. These pooled logistic model odds ratios approximate the hazard ratios (HRs) as the outcome is rare at all times [18]. 95% confidence intervals (95%CIs) were obtained using *percentiles* of 1000 bootstrap samples for the main analysis and 200 samples for stratified analyses. This method may result in asymmetric 95%CIs for risk estimates [19].

Absolute risks were estimated by fitting the pooled logistic models with interaction terms between the strategy indicator and the month of the follow-up variables. The models' predicted values were then used to estimate the cumulative incidence graphs of CRC incidence and mortality from baseline. The cumulative incidence curves were standardized to the baseline variables [20].

Because the artificial censoring required by our analytic approach can introduce selection bias due to post-baseline variables, we estimated IPWs for the outcome models. IPWs were modeled using all baseline and time-variant covariates as specified above to the model of aspirin initiation within 24 months, if the patient had not initiated aspirin previously. For the aspirin arm, we used the model to estimate the probability of initiating aspirin at 24 months if it had not been initiated previously (i.e., the probability of remaining uncensored at the last month); during the first 23 months, the probabilities were set to 1, and after month 24, the weights remained constant (ie, were multiplied by 1). For the non-initiators, we used the model to predict the probability of not initiating aspirin for months 1–24 and were similarly constant after month 24 as no further artificial censoring occurred. Weights were truncated at 99.8% to avoid heavy outlier effects. We also performed similar separate trial emulations in subgroups of individuals: men, women, young individuals (< 66 years old at diagnosis of colorectal polyps), no previous cancer diagnosis, no prior diabetes and individuals with a villous component of the baseline polyp. We also performed analyses of bleeding related cause of death as outcome (negative effect control–definition in Supplementary table 1). Further we also performed a posthoc analysis using a 12 month grace period for the outcome CRC mortality.

We used SAS 9.4 for the analyses.

The study was approved by the Stockholm Ethics Review Board.

## Results

**Fig. 1:**
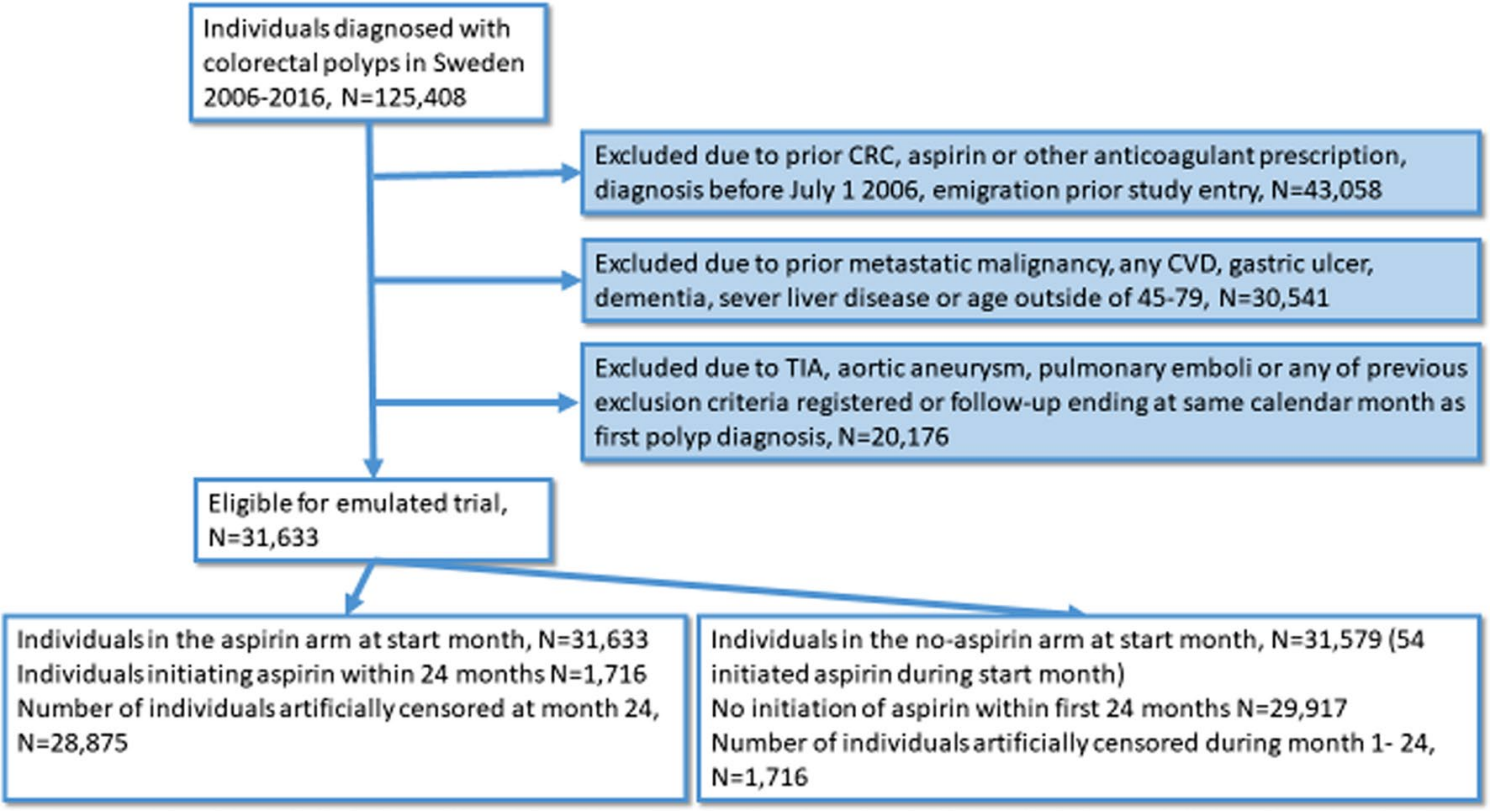
Participant flowchart. CRC, colorectal cancer. CVD, cardiovascular disease. TIA, Transient ischemic attack. The 1,042 individuals in the aspirin arm not initiating or being artificially censored reached end of follow-up within months 1–23

Of 31,633 individuals meeting the inclusion criteria, 1716 (5.4%) initiated aspirin within 2 years of polyp diagnosis (Flowchart in Fig. 1). Of those 80% had more than a year in between first and last expedition of aspirin (over 50% had more than 5 years and around 25% had more than ten years of use). The median length of follow-up was 8.07 years and the median age of the study participants was 63 years. The number of study participants, events and crude incidence rates for CRC mortality according to initiation status at 2 years and pre-specified strata are presented in Table 1. Initiation of aspirin was more common in men, older individuals and those with ≤ 9 years of education. Aspirin initiators were compared with 29,917 non-initiators.

**Table 1:** CRC mortality and incidence in Aspirin initiators and non-initiators

	Participants, n (%), total person-years		CRC mortality events, n (%)		CRC mortality rate per 1000 person-years (95%CI)		CRC incident events*, n (%)		CRC incidence* rate per 1000 person-years (95%CI)	
Group	Aspirin	No aspirin	Aspirin	No aspirin	Aspirin	No aspirin	Aspirin	No aspirin	Aspirin	No aspirin
All	1 716 (100.0%), 13 811	29 917 (100.0%), 238 501	14 (0.8%)	219 (0.7%)	1.0 (0.5–1.5)	0.9 (0.8–1.0)	109 (6.4%)	1 632 (5.5%)	7.9 (6.4–9.4)	6.8 (6.5–7.2)
Women	780 (45.5%), 6 478	16 052 (53.7%), 130 450	5 (0.6%)	113 (0.7%)	0.8 (0.1–1.4)	0.9 (0.7–1.0)	47 (6.0%)	882 (5.5%)	7.3 (5.2–9.3)	6.8 (6.3–7.2)
Men	936 (54.5%), 7 332	13 865 (46.3%), 108 050	9 (1.0%)	106 (0.8%)	1.2 (0.4–2.0)	1.0 (0.8–1.2)	62 (6.6%)	750 (5.4%)	8.5 (6.4–10.6)	6.9 (6.4–7.4)
*Age (years)*										
45–54	147 (8.6%), 1 247	5 985 (20.0%), 50 901	2 (1.4%)	20 (0.3%)	1.6 (0.0–3.8)	0.4 (0.2–0.6)	14 (9.5%)	166 (2.8%)	11.2 (5.3–17.1)	3.3 (2.8–3.8)
55–64	505 (29.4%), 4 351	11 083 (37.0%), 91 170	2 (0.4%)	54 (0.5%)	0.5 (0.0–1.1)	0.6 (0.4–0.8)	19 (3.8%)	495 (4.5%)	4.4 (2.4–6.3)	5.4 (5.0–5.9)
65–74	778 (45.3%), 6 147	10 254 (34.3%), 78 204	4 (0.5%)	104 (1.0%)	0.7 (0.0–1.3)	1.3 (1.1–1.6)	55 (7.1%)	720 (7.0%)	8.9 (6.6–11.3)	9.2 (8.5–9.9)
75–79	286 (16.7%), 2 063	2 595 (8.7%), 18 224	6 (2.1%)	41 (1.6%)	2.9 (0.6–5.2)	2.2 (1.6–2.9)	21 (7.3%)	251 (9.7%)	10.2 (5.8–14.5)	13.8 (12.1–15.5)
*Start of follow-up*										
2006–2009	696 (40.6%), 6 813	9 783 (32.7%), 101 439	5 (0.7%)	96 (1.0%)	0.7 (0.1–1.4)	0.9 (0.8–1.1)	47 (6.8%)	668 (6.8%)	6.9 (4.9–8.9)	6.6 (6.1–7.1)
2010–2013	468 (27.3%), 3 599	8 818 (29.5%), 70 376	6 (1.3%)	68 (0.8%)	1.7 (0.3–3.0)	1.0 (0.7–1.2)	38 (8.1%)	468 (5.3%)	10.6 (7.2–13.9)	6.6 (6.0–7.3)
2014–2016	440 (25.6%), 2 170	9 768 (32.7%), 48 818	1 (0.2%)	37 (0.4%)	0.5 (0.0–1.4)	0.8 (0.5–1.0)	16 (3.6%)	379 (3.9%)	7.4 (3.8–11.0)	7.8 (7.0–8.5)
*Education*										
≤ 9 years	542 (31.6%), 4 429	7 284 (24.3%), 58 852	6 (1.1%)	75 (1.0%)	1.4 (0.3–2.4)	1.3 (1.0–1.6)	42 (7.7%)	488 (6.7%)	9.5 (6.6–12.3)	8.3 (7.6–9.0)
10–12 years	700 (40.8%), 5 616	13 089 (43.8%), 104 856	3 (0.4%)	86 (0.7%)	0.5 (0.0–1.1)	0.8 (0.6–1.0)	41 (5.9%)	693 (5.3%)	7.3 (5.1–9.5)	6.6 (6.1–7.1)
> 12 years	446 (26.0%), 3 634	9 091 (30.4%), 73 438	4 (0.9%)	39 (0.4%)	1.1 (0.0–2.2)	0.5 (0.4–0.7)	25 (5.6%)	422 (4.6%)	6.9 (4.2–9.6)	5.7 (5.2–6.3)
Missing	28 (1.6%), 131	453 (1.5%), 1 353	1 (3.6%)	19 (4.2%)	7.6 (0.0–22.6)	14.0 (7.7–20.4)	1 (3.6%)	29 (6.4%)	7.6 (0.0–22.6)	21.4 (13.6–29.2)

^*^Initiators and non-initiators of Aspirin during the first 2 years after colorectal polyp

The HR (95%CI) for CRC mortality was 0.90 (0.75–1.06) for the full IPW model, 0.97 (0.75–1.06) for the baseline model and 0.97 (0.82–1.14) for the unadjusted model. In our posthoc model using a 12 months grace period corresponding numbers were 0.92 (0.77–1.60) for full model, 0.97 (0.76–1.07) baseline and 0.97 (0.82–1.15) unadjusted. The HR for CRC incidence was 0.88 (0.86–0.90) for essentially all three models (differing by < 0.02 in the CI estimates). The HR (95%CI) for all-cause mortality was 1.18 (1.12–1.24) in the full IPW model, 1.18 (1.11–1.23) in the baseline model and 1.31 (1.25–1.38) in the unadjusted model. IP weights for CRC incidence had a max value of 2003, mean = 1.81 and p99.8 = 1.28, for mortality outcomes IP weights max was 2077, mean = 1.79 and p99.8 = 1.27. In total, 18 (1.1%) of the aspirin initiators had a bleeding-related cause of death; this compares with 166 (0.6%) non-aspirin users, corresponding HR for bleeding-related cause of death were 1.45 (1.05–2.07) for the unadjusted, 1.45 (0.90–1.70) for baseline adjusted and 1.23 (0.89–1.73) for full IPW models.

**Fig. 2:**
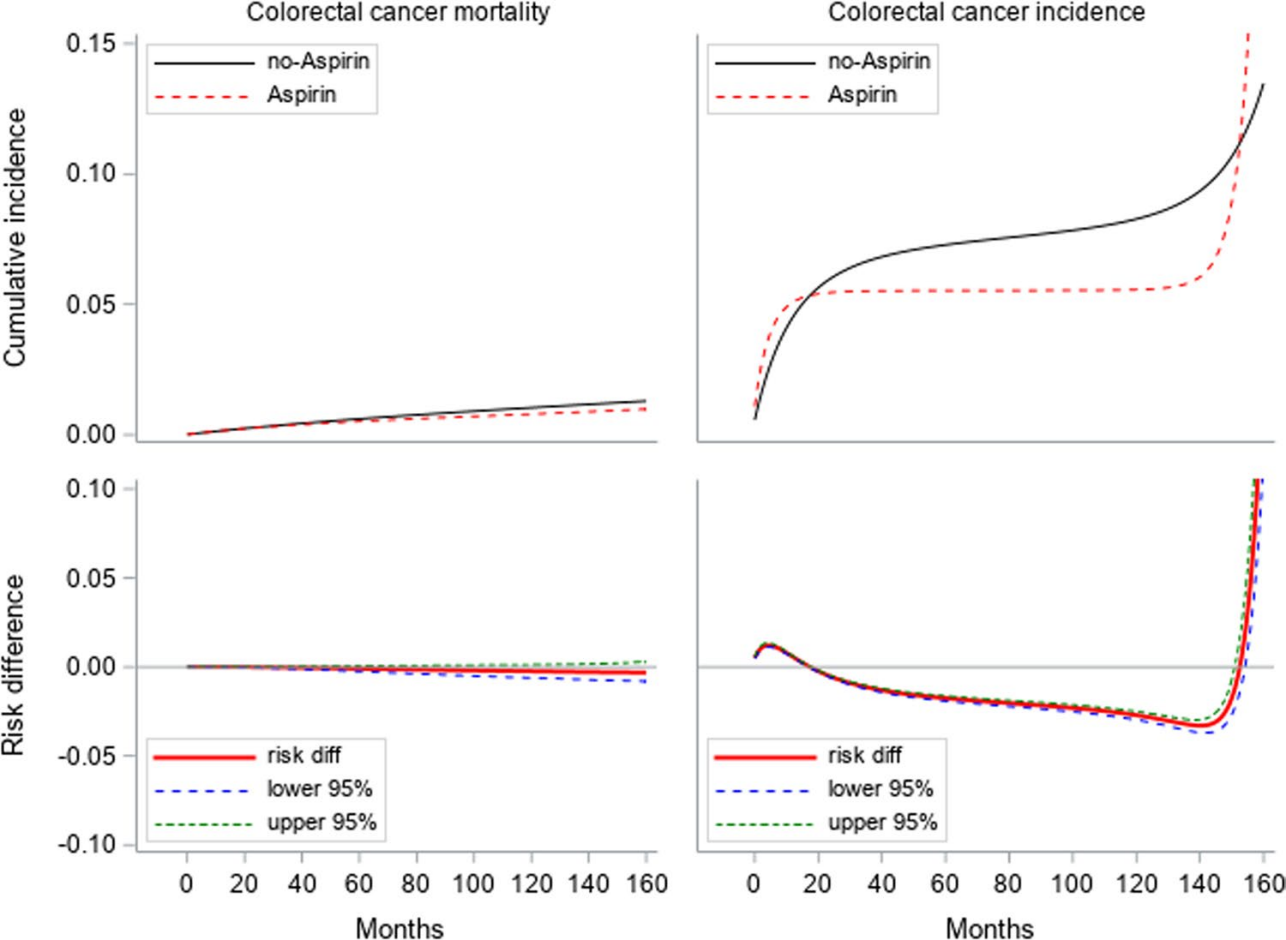
Colorectal cancer (CRC) incidence and CRC cancer mortality standardized to baseline variables

**Fig. 3:**
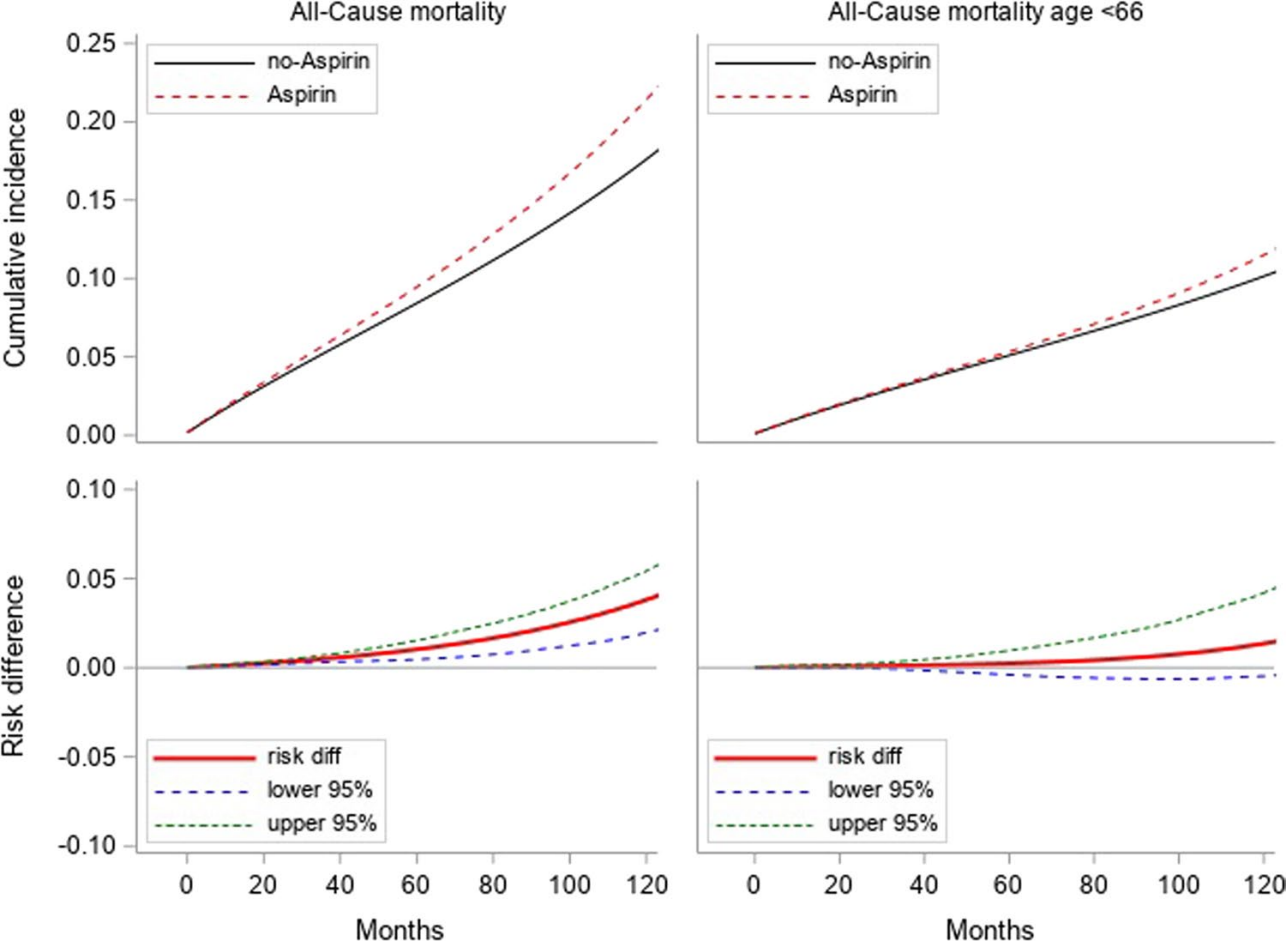
All-cause mortality in those aged < 66 years and all participants

The cumulative 10-year incidence of CRC standardized to baseline variables was about 6% in initiators and 8% in non-initiators, risk difference − 2.7% (− 3.0– − 2.5%) (Fig. 2). For CRC mortality, the 10-year cumulative incidence was 1% for both groups, risk difference − 0.2% (− 0.6 − ( +)0.1%) in our emulated models compared to crude rates of 0.8% in initiators vs. 0.7% in non-initiators for a median follow-up of 8 years (Table 1). The cumulative incidence of all-cause mortality at 10 years was 21% in initiators vs. 18% in non-initiators, corresponding risk difference 3.8% (2.0–5.4%). In individuals aged < 66 years we found that all-cause mortality increased in those who initiated aspirin, but the risk difference was smaller and confidence interval was non-significant(11% vs. 10%, risk difference 1% (− 0.05–4.2%) than that for all ages at 10-years due to fewer events (Fig. 3).

Stratified analyses (Table 2) showed that ***no subgroup*** had a significant reduction in CRC mortality. A decrease in CRC incidence was only significant in the subgroups < 66 years of age, women, villous polyps and without prior diabetes.

**Table 2:** Hazard Ratios (HRs) for CRC incidence and mortality after aspirin initiation in different subgroups

	CRC mortality, HR* (95%CI)			CRC incidence, HR* (95%CI)		
Subgroup	Unadjusted	Baseline adjusted#	Full IPW model¤	Unadjusted	Baseline adjusted#	Full IPW model¤
All	0.97 (0.82–1.14)	0.97 (0.75–1.06)	0.90 (0.75–1.06)	0.88 (0.86–0.90)	0.88 (0.85–0.89)	0.88 (0.86–0.90)
Men	0.99 (0.79–1.39)	0.99 (0.74–1.26)	0.92 (0.32–1.26)	0.89 (0.87–0.93)	0.89 (0.86–0.92)	0.90 (0.78–1.66)
Women	0.94 (0.75–1.21)	0.94 (0.72–1.17)	0.90 (0.72–1.17)	0.87 (0.85–0.90)	0.87 (0.84–0.89)	0.87 (0.84–0.90)
Age < 66 years	0.93 (0.73–1.26)	0.93 (0.67–1.20)	0.87 (0.67–1.20)	0.87 (0.85–0.90)	0.87 (0.85–0.89)	0.87 (0.85–0.90)
No previous cancer	1.05 (0.86–1.26)	1.05 (0.81–1.18)	0.98 (0.43–1.18)	0.89 (0.87–0.91)	0.89 (0.86–0.90)	0.89 (0.87–1.17)
No previous diabetes	1.00 (0.84–1.24)	1.00 (0.78–1.14)	0.93 (0.78–1.14)	0.88 (0.86–0.90)	0.88 (0.85–0.89)	0.88 (0.86–0.91)
Baseline polyp has villous component	0.98 (0.77–1.18)	0.98 (0.78–1.20)	1.00 (0.37–1.15)	0.88 (0.86–0.91)	0.88 (0.86–0.91)	0.89 (0.86–0.92)

In addition to the baseline variables also the following time-varying covariates (coded as 0 until the first month of occurrence and thereafter always 1) were used in the follw IPW model: ischemic stroke, bleeding stroke, myocardial infarction, angina pectoris, liver disease, dementia, ulcer, TIA (transient ischemic attack), heart failure, COPD, diabetes, pulmonary emboli, aneurysm, other malignancy and metastatic malignancy

*IPW* inverse probability weight

^*^HR, Hazard ratio based on pooled logistic regression for defined subgroups

^#^Adjusted for Charlson score (0, 1, 2, 3, 4, ≥ 5), number of ever pre-baseline registered visits in the Swedish Patient Registry (continuous), any diabetes, heart failure, malignancies other than CRC, diagnosis of chronic obstructive pulmonary disease (COPD, proxy for heavy smoking), year of study entry, age, sex, number of diagnosed colorectal polyps (1, 2, ≥ 3) and educational attainment (≤ 9, 10–12, > 12 years and missing)

## Discussion

Our study showed that CVD-naive individuals initiating aspirin within 2 years of polyp diagnosis had a 2% lower 10-year cumulative incidence of CRC. No difference was seen in CRC mortality. Start of aspirin use was further associated with an 18% increased relative risk of all-cause mortality and an absolute mortality risk difference of + 4% after 10 years. Because most individuals with myocardial infarction and stroke should have been prescribed aspirin at the first cardiovascular event, the increased risk of all-cause mortality may include a lack of positivity, i.e. probability of having the exposure, conditional on covariates, is greater than 0 and less than 1, for all strata and exposure levels of interest. However, we checked that positivity criteria were held in the data and also adjusted for these variables in our IPW model without more than marginal effects. Our estimated HR of 1.18 for all-cause mortality is close to the HR of 1.14 reported from the ASPREE trail [21], and also older meta-analyses of all previous primary prevention trials of aspirin including high risk CVD groups and health care workers have failed to show a beneficial effect of aspirin on all-cause mortality (HR/OR 0.94 ranging from 0.80 to 1.25 in individual studies) [22]. Therefore, it is possible that aspirin intervention may be linked to a real increase in all-cause mortality in older [23] individuals lacking manifest CVD risk factors. Another prevention study of aspirin initiation in individuals with a history of atherosclerotic risk factors but without manifest atherosclerotic disease at age 60–85 years was terminated prematurely because no beneficial effect was observed [24]. However we canont rule out that CVD in being an indication for aspirin initiation may still have caused undetectable positivity problems that impacted our all-cause estimate to be higher than a true causal estimate, but should however not have had any impact on CRC incidence and mortality. Our findings add to the accumulating evidence that aspirin has no net mortality benefit when used as a chemoprevention agent in CRC, even in individuals with increased risk of CRC without CVD risk factors at polyp diagnosis. Further we acknowledge that the results of this emulated trial should be interpreted as effects of aspirin initiation in addition to polyp removal. However for individuals with polyps detected we believe that aspirin initiation without polyp removal is not a realistic treatment alternative. Because we chose not to include individuals with a history of previous CVD events, this selection, and hence lack of baseline risk of competing CVD death, may explain some discrepancies from previous extensions of cardiovascular trials showing beneficial results [25]. However, it is impossible to perform a good observational study without excluding individuals with previous CVD, given that they are unlikely to be eligible for aspirin as chemoprevention if they did not initiate the drug for well-documented secondary prevention following a CVD event.

Our study is the first to specifically emulate a target trial of aspirin initiation in individuals with a newly diagnosed polyp. This group represents a selection of the general population expected to benefit most from CRC chemoprevention with aspirin, particularly for those with villous components (not recommended surveillance in Sweden during the study period unless the size was > 10 mm [26]) at a high risk of incident cancer [4]. Here, we found a reduced risk of incident CRC but no impact on CRC mortality; and no significant benefit in CRC mortality was found in any subgroup. On the contrary, crude/unadjusted numbers showed higher CRC mortality in the initiators (0.8% vs. 0.7%) after a median follow-up of 8 years.

Our study has several strengths, including a large cohort, high validity of the nationwide Swedish registers (National Patient Register [27], Cause of Death Register [28], Cancer Register [29], Prescribed Drug Register [15] and ESPRESSO [12]), a well-defined intervention in terms of time in relation to polyp detection, a relevant population selected to benefit the most from potential chemoprevention and the use of a robust methodology allowing for causal inference. Causal inference methods design, when data satisfy assumptions, avoid several common biases of observational studies (e.g., immortal time and prevalent user bias). In addition, it relates to a well-defined intervention that can be translated into clinical practice [30]. Finally, our median follow-up of 8 years is longer than the 4.7 years of the ASPREE trial and spans a time frame that could detect differences in long-term CRC mortality.

Our study has several limitations worth noting. We lacked information on the size and exact colonic location of polyps, smoking status (although we used COPD as a proxy measure for heavy smoking), body mass index, alcohol use, lifestyle risk factors and diet. We also lacked information on the number of endoscopic examinations during the follow-up. Hence there is a potential for residual or unmeasured confounding from the above mentioned factors as well as from lack of adjustment for other medications. The direction of this bias would likely overestimate all-cause mortality among aspirin users due to higher prevalence of risk factors of all-cause mortality among aspirin users, but how it would affect CRC incidence/mortality is not clear as CVD is also a competeing event in individuals with increasing number of combined risk factors. In this study competing events (ie. death from non CRC) was handled as a censoring event, no other analytical approach was used, however the risk of competing CVD events should be lower in this population, where previous history of such events were exclusion criteria than in studies where the population base was indeed individuals with CVD risk factors. Because most of our population was Caucasian, our results may not apply to non-Caucasian populations. At the time of the study, CRC screening in Sweden was only practiced in the capital region of Stockholm and not nationwide [31]. Therefore, our results are mainly applicable to polyps detected from symptoms. The two-year grace period was chosen to allow completion of the clinical workup for symptoms such as anemia so that any prevalent cancers were discovered during the grace period and that anemia could be reversed before initiation was mandated. We acknowledge that the effect of aspirin initiation may not be homogenous during the two year grace period (i.e. may be smaller if initiation took place 24 months vs 1 month post baseline). But as CRC is generally thought of as a slow growing cancer we do believe the average effect is representative of the overall potential clinical benefit of aspirin use. This is further supported by our posthoc analysis showing very similar results (non-significant HR = 0.92 vs 0.90) for CRC mortality using a 12 months grace period. Furthermore, we did not consider the length of aspirin use after the initial introduction, as modeling of discontinuation would cause intractable bias from discontinuation being heavily associated with shorter life expectancy, side effects or other contraindications [32]. Because aspirin initiation was slightly more common in this study's first 3.5 calendar years, we cannot exclude the possibility that some prevalent users without previously registered prescriptions in the Prescribed Drug Register were included within the first calendar period. A median follow-up of 8 years may also be too short to detect a benefit on CRC mortality.

In conclusion, this study found that in individuals with polyp removal, initiation of aspirin led to a 2% lower cumulative incidence of CRC over 10 years but had no effect on CRC mortality and was associated with a 4% higher cumulative incidence of mortality.

### Supplementary Information

Below is the link to the electronic supplementary material.

Supplementary file1 (DOCX 42 KB)

## Data Availability

No additional data available.

## Abbreviations

CI: Confidence interval
COPD: Chronic obstructive pulmonary disease
CRC: Colorectal cancer
CVD: Cardiovascular disease
DOAC: Direct oral anti-coagulant
ESPRESSO: Epidemiology Strengthened by histoPathology Reports in Sweden
GI: Gastrointestinal
HR: Hazard ratio
ICD: International Classification of Diseases
IPW: Inverse probability weight
OR: Odds ratio
SNOMED: Systematized Nomenclature of Medicine
TIA: Transient ischemic attack
USPSTF: United States Preventive Services Task Force
