# Supplementary material for: Target trial emulation of aspirin after diagnosis of colorectal polyps
Source: Eur J Epidemiol. 2023 Jun 15;38(10):1105–14. doi: 10.1007/s10654-023-01024-1 (PMC10570175; doi:10.1007/s10654-023-01024-1)
Supplement: Supplementary file 1 — Supplementary file1 (DOCX 42 KB) [file 10654_2023_1024_MOESM1_ESM.docx]

**Supplementary Materials**

**Target trial emulation of Aspirin after diagnosis of colorectal polyps**

Louise Emilsson, Mingyang Song, and Jonas F. Ludvigsson

**Supplementary Methods - Assessment of covariates**

**Supplementary Table 1.** Definition of outcome variables

**Supplementary Table 2.** International classification of disease (ICD) codes for individual comorbidities

**Supplementary Table 3.** Design of the target trial (Eligibility criteria, treatment strategies/assignment, start and end of follow-up, outcomes, statistical analysis)

**Supplementary Methods - Assessment of covariates**

We obtained data on education from the longitudinal integrated database for health insurance and labor market studies^1^, which integrates annually updated administrative information from the labor market and educational and social sectors from 1990 onward on all individuals 16 years or older registered as residents in Sweden. We used the education and income data closest to the time of polyp diagnosis. Information on age, sex, date of birth and emigration status was collected from the Swedish Total Population Register maintained by Statistics Sweden.

We calculated the Charlson comorbidity score using the SAS® macro code developed for Swedish registries^2^. We also calculated the total number of prior clinic visits before the detection of CRC for cases and their matched controls using the inpatient and outpatient records.

**References**

1. Ludvigsson JF, Svedberg P, Olen O, et al. The longitudinal integrated database for health insurance and labour market studies (LISA) and its use in medical research. *Eur J Epidemiol* 2019;34(4):423-37. doi: 10.1007/s10654-019-00511-8 [published Online First: 2019/04/01]

2. Ludvigsson JF, Appelros P, Askling J, et al. Adaptation of the Charlson Comorbidity Index for Register-Based Research in Sweden. *Clin Epidemiol* 2021;13:21-41. doi: 10.2147/CLEP.S282475 [published Online First: 2021/01/21]

**Supplementary Table 1.** Definition of outcome variables.

| Description | Relevant code | Registry used |
| --- | --- | --- |
| Incident colon cancer | ICD7: 153 | Swedish cancer registry |
| Incident rectal cancer | ICD7: 154 | Swedish cancer registry |
| Colorectal cancer mortality | ICD10: C18, C19 or C20 listed as the underlying cause of death | Swedish Cause of Death registry |
| Bleeding related cause of death | ICD10: I60, I61, I62, I64, I71, I72, I85, K25, K26 or K27 listed as the underlying cause of death | Swedish Cause of Death registry |

**Supplementary Table 2.** ICD10 codes used for exclusion criteria and covariates

| **Individual comorbidity** |  | **ICD-10 code** |
| --- | --- | --- |
| Transient ischemic attack (TIA) |  | G45 |
| Stroke (emboli) |  | I63, I64, I65, I66, I693, I694 |
| Stroke (hemorrhagic) |  | I60, I61, I62, I690, I691, I692 |
| Aneurysm |  | I67, I698 |
| Chronic obstructive pulmonary disease (COPD)* |  | J43, J44 |
| Dementia |  | F00, F01, F02, F03, F051, G30, G311, G319 |
| Diabetes (any) |  | E10, E11, E12, E13, E14 |
| Angina pectoris |  | I20, I24, I25 |
| Pulmonary emboli |  | I26 |
| Myocardial infarction |  | I21, I22, I23 |
| Heart failure |  | I110, I130, I132, I255, I420, I426, I427, I428, I429, I43, I50 |
| Gastric ulcer |  | K25, K26, K27, K28 |
| Liver disease |  | B15, B16, B17, B18, B19, K754, K746, K73, K703, K709, I850, I859, I982, I983, R18 |
| Metastatic cancer |  | C77, C78, C79, C80 |

*Used as proxy for heavy smoking

Supplementary table 3. Design of the target trial (Eligibility criteria, treatment strategies, start and end of follow-up, outcomes, statistical analysis)

| Supplementary table 3. Design of the target trial (Eligibility criteria, treatment strategies, start and end of follow-up, outcomes)  **Design of the target trial** | | | |
| --- | --- | --- | --- |
| **Inclusion criteria** | | | |
| Individuals diagnosed with any type of colorectal polyp (hyperplastic polyp, serrated, conventional, tubulovillous or villous adenoma) during July 2006-December 2016 | | | |
| Age 45-79 | | | |
| **Exclusion criteria** (any of the following registered until month of first polyp detection) | | | |
| Prescriptions of aspirin, warfarin or direct oral anticoagulant (DOAC) | | | |
| Diagnosis of CRC, dementia, metastasized malignancy, hemorrhagic stroke, gastric ulcer, aortic aneurysms, liver cirrhosis, pulmonary emboli, myocardial infarction and cerebrovascular disease including TIA | | | |
| History of previous colorectal polyp registered from 1969 and onwards | | | |
| (End of follow up occurring same month as polyp detection – only for the emulated trial*) | | | |
| **Treatment strategies** | | | |
| 1. (replicate in emulated trial*) | Aspirin initiation (prescription of >13 days of 75 mg (87% in emulate trial*) or 160 mg (13% in emulated trial*) daily) within any of the first 24 months (individuals censored at month 24 if no aspirin initiation was registered) | | |
| 1. (replicate in emulated trial*) | No aspirin initiation within the first 24 months (individuals censored from actual month of aspirin initiation registration – i.e. any of months 1-24 during the trial) | | |
| **Treatment assignment:** At baseline | | | |
| **Start of follow-up**: month of polyp detection | | | |
| **End of follow-up**: first month of outcome, death, emigration or administrative end of follow-up (Dec, 2019) | | | |
| **Outcomes** | | | |
| CRC incidence | | CRC mortality | All-cause mortality |
| **Causal contrast:** Per protocol analysis of initiation of aspirin within 24 month vs. no-initiation within 24 months. | | | |
| **Statistical analysis:** Survival modelled by pooled logistic regression adjusted for covariates# and inverse probability weighting when deviation from assigned strategy. | | | |

*Differences in emulated trial vs. target trial.
